# An Intronic Heterozygous *SYNE2* Splice Site Mutation: A Rare Cause for Myalgia and hyperCKemia?

**DOI:** 10.3390/muscles3010010

**Published:** 2024-03-15

**Authors:** Theresa Paulus, Natalie Young, Emily Jessop, Carolin Berwanger, Christoph Stephan Clemen, Rolf Schröder, Rafal Ploski, Christian Hagel, Yorck Hellenbroich, Andreas Moser, Iakowos Karakesisoglou

**Affiliations:** 1 Department of Neurology, University of Lübeck, Ratzeburger Allee 160, 23538 Lübeck, Germany; t.paulus@uni-luebeck.de (T.P.); andreas.moser@uni-luebeck.de (A.M.); 2 Department of Biosciences, Durham University, South Road, Durham DH1 3LE, UK; natalie.young@durham.ac.uk (N.Y.); emilykjessop1@gmail.com (E.J.); 3 Institute of Aerospace Medicine, German Aerospace Center, Linder Höhe, 51147 Cologne, Germany; carolin.berwanger@uni-koeln.de (C.B.); christoph.clemen@uni-koeln.de (C.S.C.); 4 Center for Physiology and Pathophysiology, Institute of Vegetative Physiology, Medical Faculty, University of Cologne, Robert-Koch-Straße 39, 50931 Cologne, Germany; 5 Institute of Neuropathology, University Hospital Erlangen, Friedrich-Alexander University Erlangen-Nürnberg, Schwabachanlage 6, 91054 Erlangen, Germany; rolf.schroeder@uk-erlangen.de; 6 Department of Medical Genetics, Medical University of Warsaw, Pawińskiego 3c, 02-106 Warsaw, Poland; rploski@wp.pl; 7 Institute of Neuropathology, University Medical Center Hamburg-Eppendorf, Martinistraße 52, 20251 Hamburg, Germany; hagel@uke.de; 8 Institute of Human Genetics, University of Lübeck, Ratzeburger Allee 160, 23538 Lübeck, Germany; yorck.hellenbroich@uksh.de

**Keywords:** emerin, lamin A/C, laminopathies, LINC complex, myalgia, nesprin, nesprin-2 giant, nuclear shape, *SYNE2*

## Abstract

*SYNE2* mutations have been associated with skeletal and cardiac muscle diseases, including Emery-Dreifuss muscular dystrophy (EDMD). Here, we present a 70-year-old male patient with muscle pain and elevated serum creatine kinase levels in whom whole-exome sequencing revealed a novel heterozygous *SYNE2* splice site mutation (NM_182914.3:c.15306+2T>G). This mutation is likely to result in the loss of the donor splice site in intron 82. While a diagnostic muscle biopsy showed unspecific myopathological findings, immunofluorescence analyses of skeletal muscle and dermal cells derived from the patient showed nuclear shape alterations when compared to control cells. In addition, a significantly reduced nesprin-2 giant protein localisation to the nuclear envelope was observed in patient-derived dermal fibroblasts. Our findings imply that the novel heterozygous *SYNE2* mutation results in a monoallelic splicing defect of nesprin-2, thereby leading to a rare cause of myalgia and hyperCKemia.

## 1. Introduction

Nesprins (nuclear envelope spectrin repeat proteins) are a family of multi-isomeric signalling scaffold proteins [1,2] that are encoded by four synaptic nuclear envelope genes (*SYNE1-4*) [3,4]. They are a core component of the LINC (Linker of Nucleoskeleton and Cytoskeleton) complex, which is located at the nuclear envelope (NE) [2,5], providing a functional and stable physical connection between the nuclear interior and the cytoskeleton [2,6,7,8]. LINC participates in many fundamental cellular activities, including nuclear morphology maintenance and nuclear positioning during cell migration and differentiation, during which it regulates mechanical cell signalling and gene expression [2]. An intact LINC complex, containing nesprins, is critical for muscle cell differentiation and development, as well as for maintaining muscular physiological functions [2,5].

Nesprin-2 giant is encoded by *SYNE2* and constitutes the third largest human protein (~800 kDa) [2]. There are additional nesprin-2 isoforms that are structurally and functionally diverse due to extensive alternative transcription and splicing of the gene [2,6,7]. Nesprin-2 giant comprises an N-terminal paired Calponin Homology (CH) domain that links the nucleus to F-actin, and a C-terminal Klarsicht/ANC-1/Syne Homology (KASH) domain that traverses the outer nuclear membrane (ONM) and interacts with SUN proteins in the inter-membrane space. These domains are linked by the central rod domain, which contains a variable number of spectrin repeats (SR) and mediates protein–protein interactions [2,4,8,9]. Nesprins localise to multiple subcellular compartments including the NE, nucleoplasm, and cytoplasm [1,7,9], and are essential for the structural integrity of chromatin, the nucleoskeleton, and cytoskeleton, as well as for proper signal transduction across the NE [10,11,12].

Nesprin-2 is highly expressed in cardiac and skeletal muscles [2,4,13] and is involved in their pathology, including Emery-Dreifuss muscular dystrophy (EDMD) and dilated cardiomyopathy (DCM) [2,3,5]. EDMD is a neuromuscular disorder characterised by progressive skeletal muscle wasting and a potentially fatal cardiomyopathy. Although mutations in emerin (EDMD1) and lamin A/C (EDMD2) lead to EDMD or EDMD-like phenotypes, these phenotypes can also be caused by nesprin-1 or nesprin-2 mutations [13]. Therefore, as lamin A/C and emerin mutations cause similar diseases to nesprin mutations, this strengthens the view that LINC complex perturbations are the critical step leading to muscle disease [2,13]. To date, it is unknown how exactly nesprin mutations cause muscle dysfunction and which of the multiple known functions of the nesprins are key to muscle development and physiology [4,13]. In this case report, we describe a male patient with myalgia and hyperCKemia, in whom we identified a novel *SYNE2* splice site mutation.

## 2. Results

### 2.1. Adult Patient Case with Myalgia and hyperCKemia

#### 2.1.1. Patient Observations and Family History

A 70-year-old male patient presented in March 2021 to the neurological outpatient clinic of the University Hospital Schleswig-Holstein (UKSH), Lübeck, with a 3-year history of slowly progressive muscle pain in the proximal lower limbs that increased with physical activity. Walking distance was limited (<500 m), and gait deficits, which occurred after cerebral infarction with manifested Wallenberg’s syndrome in 2012, worsened. HMG-CoA reductase inhibitor (statin) treatment was terminated in 2014. Further, numbness and night-time burning of the feet was reported. Besides cerebral infarction, the case history included arterial hypertension and obesity as pre-existing conditions. Intellectual aspects remained normal until now. He did not complain of any cardiac symptoms. The patient reported no alcohol consumption. Chronic muscle diseases in his family were not known to him. The patient’s siblings, two younger brothers, and the patient’s children, one daughter and one son, are all healthy. His mother, 93 years old, also has no muscle disease signs. His father died due to Alzheimer’s and Parkinson’s disease at the age of 77 years.

#### 2.1.2. Clinical Examination

An externally performed lumbar MRI in 2019 revealed degenerative changes and facet joint degeneration, leading to affection of the L3/4 nerve root—however, without pain or any neurological deficits. Neurological examination showed mild left Wallenberg’s syndrome and a mild sensible distal symmetric neuropathy with impaired vibration sense. Walking on his heels or toes was very unstable. There were neither signs of manifested limb weakness, myotonia, nor evidence of myasthenia gravis. Laboratory tests showed elevated serum creatine kinase (CK) levels (1355 U/L, normal <190 U/L). Routine laboratory screenings, including thyroid function, Vitamin B12, and folic acid, were within normal range.

Further investigations were performed during March/April 2021. Myography of the left deltoid and vastus lateralis muscles presented no spontaneous activity, and sensory and motor neurography (left sural nerve, right tibial nerve) did not reveal significant findings. MRI scans of the upper legs indicated a normal signal intensity within skeletal muscles without evidence of muscle atrophy. Cerebrospinal fluid analysis revealed a normal cell count (1 cell/μL). CK was elevated (289 U/L), with a slight increase after physical activity (352 U/L); the CK values remained elevated during follow-up visits. Ischemic lactate-ammonia test revealed no pathologic findings. Furthermore, the patient did not report any worsening of symptoms but expressed that they caused severe impairment in everyday life. Prednisolone treatment did not yield a significant improvement of the symptoms. Therefore, the steroid therapy was gradually tapered off.

#### 2.1.3. Genetic Analysis

**Figure 1:**
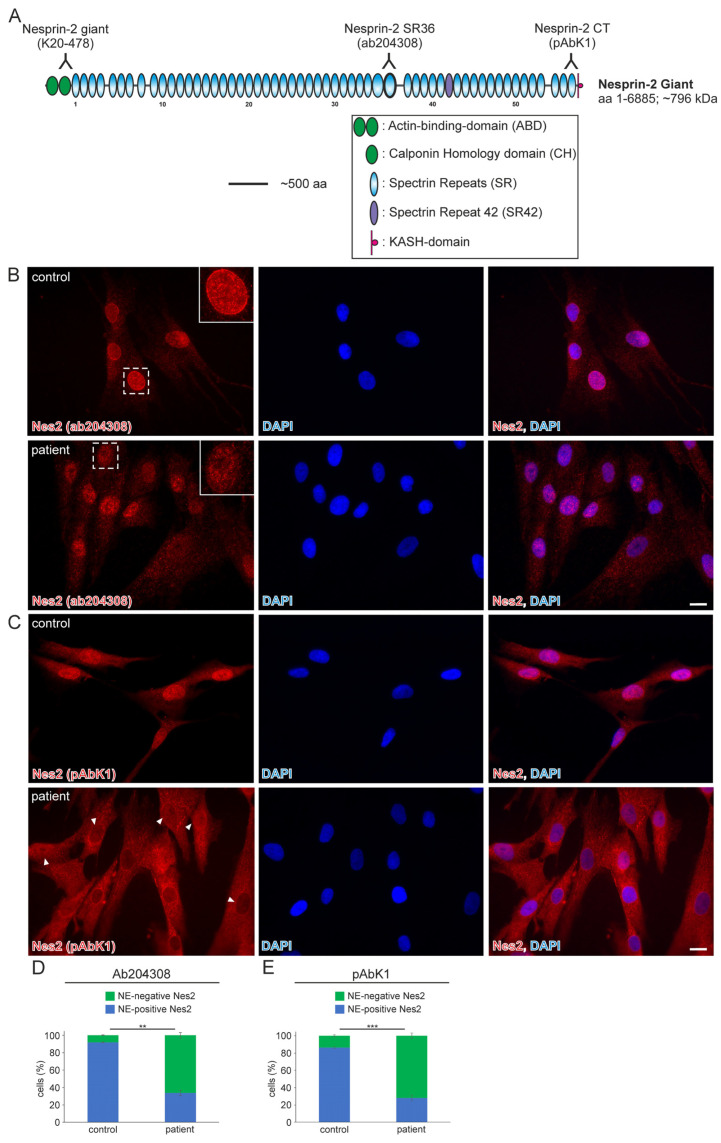
The nuclear envelope nesprin-2 localisation in *SYNE2* patient dermal fibroblast cells is compromised. (**A**) Nesprin-2 giant schematic diagram. Nesprin-2 domains are colour-coded. Actin-binding-domain (ABD, green); Spectrin repeats (SR, blue); spectrin repeat 42 (SR42, purple); KASH-domain (red). Relative positions (inverted Y) of the nesprin-2 antibodies are indicated above the nesprin-2 giant protein schematic. (**B**) Immunofluorescence examination of control and patient dermal fibroblast cells using the nesprin-2 (Nes2) ab204308 antibody. Insets are higher magnifications of boxed areas. DAPI is used to counterstain nuclei. Scale bar: 20 μm. (**C**) Immunofluorescence of control and patient cells using the nesprin-2 (Nes) pAbK1 antibody. DAPI denotes nuclei. Arrowheads highlight patient nuclei where either nesprin-2 stain is lacking, discontinuous, or weak. Scale bar: 20 μm. (**D**,**E**) Graphs highlighting the nesprin-2 ab204308 antibody (**D**) and nesprin-2 pAbK1 antibody (**E**) staining pattern in control and patient cells. Error bars represent the Standard Error of the Mean (SEM). Statistical evaluation was conducted using an unpaired Student’s *t*-test, *p* < 0.01 (**), *p* < 0.001 (***).

Whole-exome analysis revealed the heterozygous variant NM_182914.3:c.15306+2T>G in *SYNE2* (ClinVar database: Variation ID 1710169, accession VCV001710169.2). This variant is a thymine (**T**) to guanine (G) transversion located at the second nucleotide (+2) of the 5′-splicing donor site of intron 82 (Ensembl genome data base; transcript ENST00000358025.7; encoded by 116 exons). The deviation from the canonical G**T**-AG (5′-donor and 3′-acceptor dinucleotides) mRNA-splicing rule most likely results in the loss of the donor splice site of *SYNE2* intron 82. The mutation was classified as a variant of uncertain significance and has not previously been described in the literature. The *SYNE2* exons (82 and 83) that flank the mutated intron encode for nesprin-2 giant spectrin repeat 42 (SR42), which is closer to the C-terminal nuclear membrane anchoring KASH-domain than the N-terminal actin-binding-domain (Figure 1A).

#### 2.1.4. Muscle Biopsy Findings and Molecular and Cell Biology Examinations

**Figure 2:**
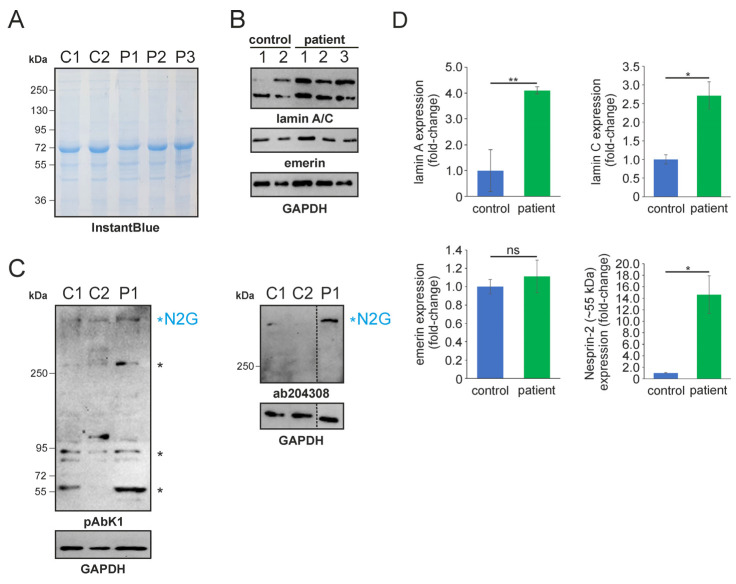
SDS-PAGE and Western blotting analysis of human control and patient dermal fibroblast cells. (**A**) SDS-PAGE of control (C1-2) and patient (P1-3) fibroblast lysates. Lysate numbers highlight different technical repeats. Gel counterstained with InstantBlue to indicate equal protein loading. (**B**) Western blot analysis of control and patient cell lysates using lamin A/C, emerin, and GAPDH (highlighting equal protein loading) antibodies. (**C**) Nesprin-2 protein Western blot analysis using the pAbK1 (left) and ab204308 (right) reagents. Major nesprin-2 isoforms (including nesprin-2 giant [N2G]) are highlighted with asterisks. The discontinued vertical line highlights that the Western blot signal for the patient sample (P1) was digitally cropped and re-assembled from the same gel. Ab204308 detects N2G protein only, while pAbK1 detects N2G and other smaller nesprin-2 isoforms. (**D**) Lamin A/C, emerin, and nesprin-2 (~55 kDa) expression levels in control and patient cells. The error bars represent the Standard Error of the Mean (SEM). Statistical evaluation was conducted using an unpaired Student’s *t*-test. The significance levels are indicated as follows: *p* < 0.05 (*), *p* < 0.01 (**), and non-significant (ns).

**Figure 3:**
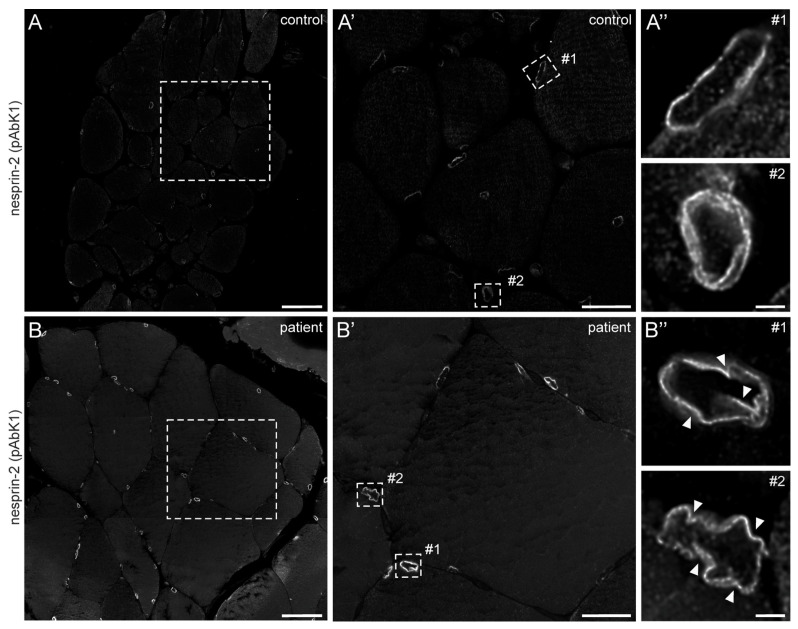
Nesprin-2 antibody staining of human control and patient muscle biopsies. Confocal microscopy of nesprin-2 (pAbK1) stained control (**A**) and patient (**B**) muscle tissue paraffin sections. Dashed boxes in (**A**,**B**) are shown magnified in panels (**A′**,**B′**), respectively. Panels (**A″**,**B″**) are deconvolved images of myonuclei (#1) and endomysial nuclei (#2) shown in (**A′**,**B′**) (dashed rectangles), respectively. Arrowheads denote nuclear deformations. Microscopy settings are identical for panels (**A**,**B**,**A′**,**B′**,**A″**,**B″**). Scale bar: 50 μm (**A**,**B**), 20 μm (**A′**,**B′**) and 2 μm (**A″**,**B″**).

In 2022, a left vastus lateralis muscle biopsy and human fibroblasts cultured from skin biopsy [14] were both acquired from the patient. Muscle biopsy examination showed mild and unspecific myopathological alteration, comprising a few atrophic muscle fibers, some fibers with internalised nuclei (Supplementary Figure S1), and a single fiber necrosis. Immunohistochemical stainings revealed the appropriate localisation of lamin A/C and emerin at the nucleus (Supplementary Figure S1). Next, immunofluorescence (IF) and Western blotting (WB) analyses of muscle and dermal cells were executed to analyse mutational effects at the protein level. IF analysis of human control and patient dermal fibroblast cells showed that the nuclear envelope nesprin-2 localisation in *SYNE2* patient dermal fibroblast cells is compromised with the use of three independent nesprin-2 antibodies (Figure 1A), the ab204308 (Figure 1B), the pAbK1 (Figure 1C), and the K20-478 antibody (Supplementary Figure S2). The pattern of nesprin-2 ab204308 and pAbK1 antibody staining in control and patient cells was quantified, revealing a significantly increased number of cells with nesprin-2-negative nuclei in the patient compared to controls (Figure 1D,E). Further, IF analysis showed significantly higher levels of nuclear shape irregularities in patient dermal fibroblast cells compared to control fibroblasts (Supplementary Figures S3 and S4). Emerin, lamin A/C, and lamin B1 localisation, however, were not compromised in patient dermal fibroblast cells (Supplementary Figure S4). The WB results from human control dermal fibroblast cells and patient dermal fibroblast cells showed higher expression levels of lamin A/C, nesprin-2 isoforms (especially nesprin-2 55 kDa isoform) (Figure 2), and nesprin-2 giant (Supplementary Figure S5) in patient cells. However, WB analysis of lamin B1 levels, a biomarker for ageing, did not reveal any significant alterations in protein expression (Supplementary Figure S6). Additionally, no noticeable effects on emerin expression were observed (Figure 2). In contrast to the dermal fibroblast cells, IF analysis of the control and patient muscle tissue (Figure 3) did not reveal any obvious difference in nuclear envelope nesprin-2 localisation. However, the nesprin-2 staining intensity was stronger in the patient sample, and there were pronounced nuclear shape irregularities in the patient compared to control muscle fibers and endomysial fibroblasts.

## 3. Discussion and Conclusions

Here, we analysed the consequences of a novel heterozygous *SYNE2* splice site mutation that we identified in a male patient with myalgia and hyperCKemia. In patient-derived dermal fibroblasts, we found less nesprin-2 localising to the NE compared to control cells, using three distinct antibodies that cover the entire length of the nesprin-2 giant molecule. In patient muscle, however, nesprin-2 protein was clearly detected at the nucleus. This highlights phenotypic differences between proliferating fibroblasts and post-mitotic skeletal muscle tissues. Most likely, these differences arise due to the dominant expression of nesprin-2 giant in fibroblasts compared to mature skeletal muscle. During myogenesis, nesprin-1 and nesprin-2 transition from the giant isoforms to the smaller variants, which become predominant in mature muscle [15,16]. Further, we demonstrated that patient fibroblasts and muscle fibers showed significantly higher levels of nuclear shape irregularities compared to control cells. This is in accordance with other described *SYNE2* mutations, which have also been shown to lead to NE malformations and an altered nuclear morphology [2,17].

Expression of a severely truncated nesprin-2 mutant protein variant due to a frameshift (intron inclusion) was not found in patient cells. Thus, *SYNE2* transcript expression seems not to be affected by the mutation. Further analyses in this study have shown higher expression levels of lamin A/C and nesprin-2 isoforms (especially nesprin-2 55 kDa isoform) in patient dermal fibroblast cells compared to control cells. This could possibly be caused by differences in the extracellular environment of the patient and control subjects. The patient might have a more rigid extracellular matrix, leading to higher expression levels of lamin A/C and nesprin-2 [18]. Alternatively, the changes in protein expression may be a compensatory effect due to the potential removal of some key nesprin-2 giant domains, including SR42 (e.g., exon-skipping), and the abnormal transmission of forces to the nuclear lamina.

*SYNE2* mutations and impairment of nesprin-2 functions, including their essential role for the structural integrity of chromatin, the nucleoskeleton, and cytoskeleton as well as for proper signal transduction across the NE [10,11,12], are associated with the development of Emery-Dreifuss muscular dystrophy type 5 (EDMD5) [2,3,19]. EDMD5 is characterised by skeletal muscle weakness without obvious contractures and cardiomyopathy with arrhythmia and heart failure [19], but it is known that the clinical expression of the phenotype in EDMD and clinical phenotypes of *SYNE2* mutations are diverse [2,3,5,8,10]. Skin fibroblasts from patients with EDMD or EDMD-like phenotypes and *SYNE2* mutations showed nuclear shape abnormalities. Using immunofluorescence, altered nesprin-2 localisation was demonstrated in EDMD fibroblasts with a reduction of nesprin-2 NE staining. Further, emerin and SUN2 mislocalisation and disrupted nesprin/emerin/lamin binding interactions were found in fibroblasts from EDMD patients with *SYNE* mutations. In skeletal muscle tissue of EDMD patients, loss of nesprin from the NE was observed [3].

The patient described in this case report has symptoms of mild muscle problems starting in late adulthood; however, no cardiac symptoms were present. The described findings in the fibroblasts and muscle cells of the reported patient are alterations that have also been reported in EDMD patients with *SYNE2* gene mutations [3]. Furthermore, two children harbouring the same *SYNE2* mutation are mentioned in the Department of Medical Genetics Warsaw Medical University database. Since these children were not reported to have shown signs of skeletal muscle involvement, it is unlikely that the described gene variant has a high penetrance for causing disease symptoms at an early age.

In conclusion, we have identified a novel heterozygous *SYNE2* splice site mutation that leads to a reduced nesprin-2 giant nuclear envelope localisation, nuclear deformations in cultured dermal fibroblasts, and deformation of myonuclei and fibronuclei in skeletal muscle tissue. Thus, the mutation-inflicted splice site defect of one *SYNE2* allele seems to be the likely cause of myalgia and hyperCKemia in the reported patient.

## 4. Materials and Methods

### 4.1. Fibroblast Cultures

Human control, male, single donor, neonatal foreskin dermal fibroblast cells (Sigma-Aldrich, Gillingham, UK) and patient dermal fibroblast cells were maintained in DMEM medium containing 4.5 g/L Glucose, L-Glutamine and phenol red (Lonza, Slough, UK), supplemented with 10% Fetal Bovine Serum (FBS) and 1% Penicillin/Streptomycin (Merck, Gillingham, UK). Cells were cultured in T75 cm^2^ flasks (CytoOne, Starlab, Milton Keynes, UK) and incubated in a humidified atmosphere of 5% CO_2_ at 37 °C. We also analysed two additional primary dermal fibroblasts sourced from healthy, young male donors (one young adult and one child donor). However, because the results from these controls were consistent, we decided to present only one control group (i.e., neonatal dermal fibroblasts) in our findings.

### 4.2. Immunofluorescence (IF) of Cells and Skeletal Muscle Paraffin Sections

Control and patient dermal fibroblast cells were seeded onto high-precision glass-coverslips (number 1.5H; Marienfeld Superior, Lauda-Königshofen, Germany) and allowed to adhere for 24 h. After 24 h, cells were rinsed with PBS (Phosphate-buffered saline; ThermoFisher Scientific, Loughborough, UK) and then fixed with 4% formaldehyde/PBS solution for 20 min at room temperature (RT). After fixation, coverslips were rinsed once with PBS before being permeabilised with 0.5% Triton X-100/PBS solution for 10 min and then blocked for 1 h in PBG (1% BSA, 0.1% Fish gelatin solution in PBS; Sigma-Aldrich, Gillingham, UK). Coverslips were incubated with primary antibodies (Table S1) diluted in PBG for 2 h in a humidified chamber, followed by three PBS washes (5 min each), and then stained with the secondary antibodies (Table S1) diluted in PBG for 1 h at RT in the dark, followed by three PBS washes (5 min each). Coverslips were then incubated with a 2 μg/mL 4,6-diamino-2-phenylindone [DAPI; Sigma-Aldrich, Gillingham, UK; Table S2]/PBS solution for 5 min at room temperature in the dark to stain the DNA of nuclei. Finally, coverslips were washed three times with PBS (5 min each) before they were mounted onto glass slides using anti-fade mounting medium Vectashield H-1000 (Vector Laboratories, Peterborough, UK). Fluorescence imaging was performed using a Zeiss Axioscope 40 upright fluorescence microscope mounted with a Zeiss AxioCam MRm camera (Carl Zeiss Microscopy Deutschland GmbH, Oberkochen, Germany), and live cell imaging was performed under phase contrast using an EVOS XL Core system (ThermoFisher Scientific, Loughborough, UK).

Control and patient skeletal muscle paraffin sections were deparaffinised and incubated in citrate buffer (0.01 M, pH 6.0) at 97 °C for 20 min for epitope retrieval. Non-specific binding sites were blocked by incubating the sections in blocking buffer (5% horse serum, 1% BSA in PBS) at RT for 1 h. Sections were incubated with primary antibodies (Table S1) diluted in blocking buffer for 1 h at RT. After two washes with PBS containing 0.5% Triton X-100 and four washes with PBS (10 min each), sections were incubated with the secondary antibody (Table S1) diluted in blocking buffer. After the same six washing steps, sections were rinsed once with ddH_2_O and embedded in Mowiol/DABCO. Fluorescence images were acquired with an Infinity Line system (Abberior Instruments GmbH, Göttingen, Germany) using the UPLXAPO60XO NA1.42 objective and Imspector software version 16.3.16100 in LightBox mode, and the confocal images of single nuclei were deconvolved using Huygens Essential version 23.04.0p4 (Scientific Volume Imaging B.V., Hilversum, The Netherlands).

### 4.3. Western Blotting (WB) Analysis

Adherent control and patient dermal fibroblast cells were washed once with ice-cold PBS. After PBS aspiration, RIPA lysis buffer (50 mM Tris, 150 mM NaCl, 0.1% SDS, 1% Nonidet P-40, 0.5% Sodium-deoxycholate, 1% Protease Inhibitors (Protease Inhibitor Cocktail P2714, Sigma-Aldrich, Gillingham, UK)) was added, and the samples were incubated for 15 min on ice. Cell lysates were collected using a cell scraper (Corning, Flintshire, UK), and samples were sonicated at 40 kHz for 5 min (Branson 1510) and finally spun at 13,000× *g* for 10 min at 4 °C. The supernatant was collected, and the protein concentration was determined using a BCA Protein Assay kit (ThermoFisher Scientific, Loughborough, UK). The supernatant was mixed in a 5:1 ratio with sample loading buffer (5× Laemmli buffer, containing 5% 2-mercaptoethanol [Sigma-Aldrich, Gillingham, UK]) and incubated at 99 °C for 4 min. Proteins were analysed either through a 10% SDS-PAGE gel or a NovexTM 4–12% Tris-Glycine gradient gel (Invitrogen, Loughborough, UK). Following SDS-PAGE, gels were stained with Expedeon InstantBlue protein stain (ThermoFisher Scientific, Loughborough, UK) to evaluate both the quality of the cell lysates and equal protein loading. Protein transfer to PVDF membranes (Immobilon-P, Merck Millipore, Burlington, MA, USA) was executed as previously described in Young et al., 2021 [5]. Subsequently, PVDF membranes were incubated in primary antibodies (Table S1; diluted in 1% milk/PBS) overnight at 4 °C. Membranes were then washed three times with 0.1% Tween 20/PBS solution (pH 7.4; 10 min each) and then incubated with the secondary horseradish peroxidase-conjugated antibody (Table S1) for 1 h at RT. PVDF membranes were washed three times in 0.3% Tween 20/PBS buffer (pH 7.4; 5 min each), followed by three final washing steps in 0.1% Tween 20/PBS solution (pH 7.4; 5 min each). Finally, Clarity Western ECL Substrate (BioRad, Watford, UK) was added directly onto the membrane and incubated for 5 min in the dark, and the membranes were imaged using the iBright 1500 imaging system (ThermoFisher Scientific, Loughborough, UK), as outlined in Young et al., 2021 [5].

## Data Availability

The data presented in this study are available upon request from the corresponding author.

## Supplementary Materials

The following supporting information can be downloaded at: https://www.mdpi.com/article/10.3390/muscles3010010/s1, Figure S1: Muscle pathology of the patient; Figure S2: The nuclear envelope nesprin-2 giant protein localisation in *SYNE2* patient dermal fibroblast cells is compromised; Figure S3: Patient dermal fibroblast cells show higher level of nuclear shape defects; Figure S4: The localisation of emerin, lamin A/C, and lamin B1 in patient dermal fibroblast cells is not compromised; Figure S5: Nesprin-2 giant expression levels are increased in *SYNE2* patient fibroblast cells; Figure S6: The expression of lamin B1 is similar in control and patient fibroblast cells. Table S1: Primary and secondary antibodies used for immunofluorescence staining (IF) and Western blotting (WB); Table S2: Fluorescent stains and conjugates used for immunostaining.
