# Supplementary material for: An Intronic Heterozygous SYNE2 Splice Site Mutation: A Rare Cause for Myalgia and hyperCKemia?
Source: Muscles. 2024 Mar 15;3(1):0. doi: 10.3390/muscles3010010 (PMC12225326; doi:10.3390/muscles3010010)
Supplement: Supplementary file 1 [file muscles-03-00010-s001.zip › muscles-2801310-supplementary.pdf]

muscles

MDPI

Case Report

An Intronic Heterozygous *SYNE2* Splice Site Mutation: A Rare Cause for Myalgia and hyperCKemia?

Supplementary:

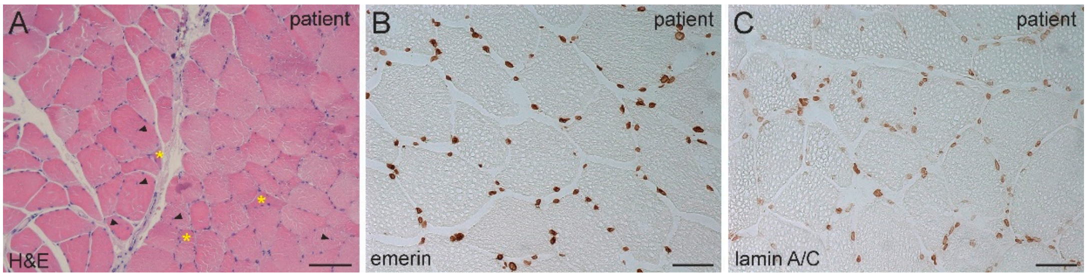

Figure S1. Muscle pathology of the patient. (**A**) Hematoxylin and eosin stain (H&E). Black arrowheads denote central nuclei in muscle fibers. Note the presence of few atrophic fibers (yellow asterisks). (**B**) Immunohistochemistry for emerin. (**C**) Immunohistochemistry for lamin A/C. Scale bar corresponds to 100 µm in the H&E photograph and to 50 µm in the immunohistochemical stains.

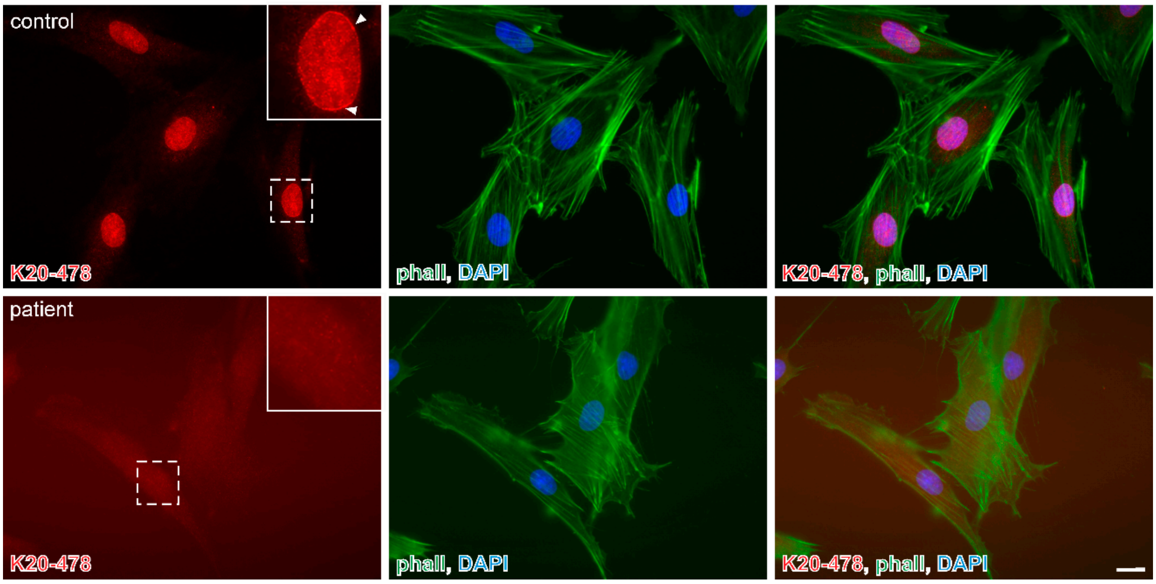

Figure S2. The nuclear envelope nesprin-2 giant protein localisation in *SYNE2* patient dermal fibroblast cells is compromised. Immunofluorescence examination of control and patient dermal fibroblast cells using the anti-nesprin-2 giant protein (N2G) K20-478 monoclonal antibody. Inset is higher digital magnification of dashed area. Arrowheads highlight the presence of N2G protein in control nuclei. FITC-conjugated phalloidin (phall) is used to visualise the F-actin cytoskeleton and the overall morphology of the studied cells. Nuclei are stained with DAPI. Scale bar 20 µm.

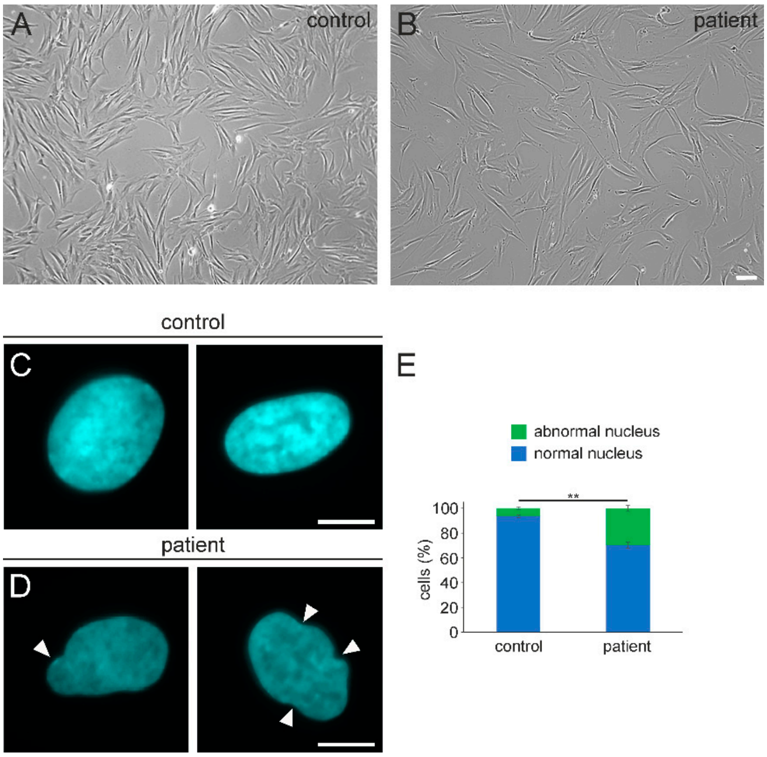

Figure S3. Patient dermal fibroblast cells show higher level of nuclear shape defects. (**A, B**) Phase contrast microscopy of control (**A**) and patient (**B**) dermal fibroblast cells. Scale bar 100 µm. (**C, D**) Immunofluorescence of control (**C**) and patient (**D**) cell DAPI-stained nuclei. Defects in nuclear shape in patient fibroblasts are indicated with arrowheads (panel **D**). Scale bars 10 µm. (**E**) The % of control and patient fibroblast cells with normal and abnormally shaped nuclei is indicated. The error bars denote the Standard Error of the Mean (SEM). Statistical evaluation using an unpaired Student's t-test, $p<0.01$ (**).

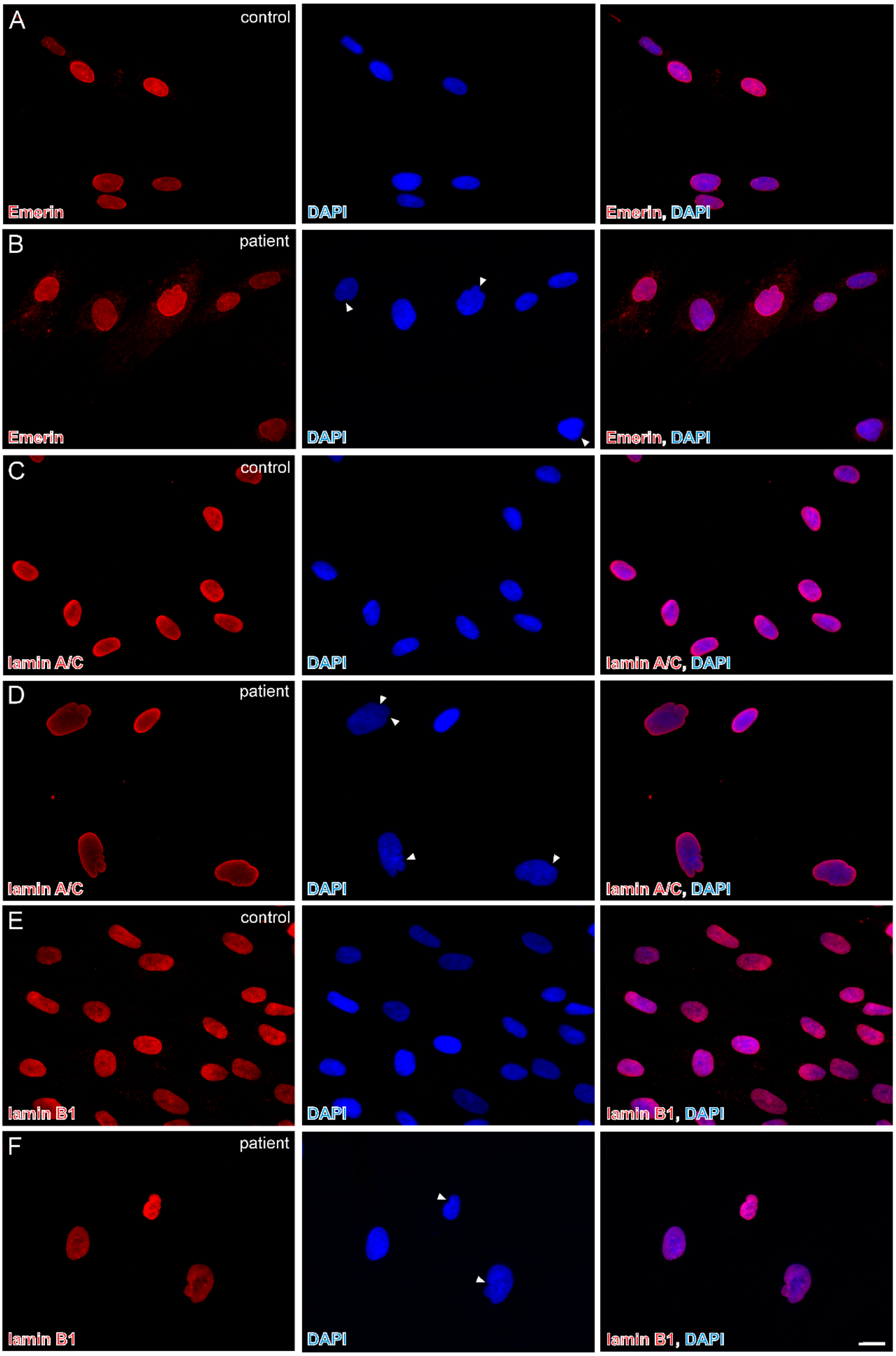

Figure S4. The localisation of emerin, lamin A/C, and lamin B1 in patient dermal fibroblast cells is not compromised. Immunofluorescence analysis of control and patient dermal fibroblast cells using anti-emerin (panels **A, B**), anti-lamin A/C (panels **C, D**) and anti-lamin B1 (panels **E, F**) antibodies. Note that all the antibodies yield similar staining in both control and patient cells. Irregularities in nuclear shape in patient cells are denoted with arrowheads. Nuclei are counterstained with DAPI. Scale bar 20 µm.

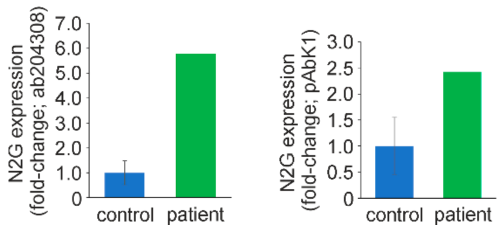

Figure S5. Nesprin-2 giant expression levels are increased in *SYNE2* patient fibroblast cells. Nesprin-2 giant (N2G) expression levels in control and patient cells using the ab204308 (left graph) and pAbK1 (right graph) rabbit polyclonal antibodies. Error bars for control denote the Standard Error of the Mean (SEM).

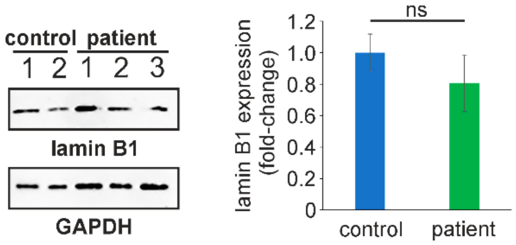

Figure S6. Lamin B1 expression is similar in control and patient fibroblast cells. A Western blot analysis was conducted to assess lamin B1 protein expression in control and *SYNE2* patient dermal fibroblasts. Lysate numbers highlight different technical repeats (left panel). GAPDH was used to verify equal loading of samples. The graph (right panel) illustrates that the observed changes in lamin B1 expression were not statistically significant (ns). The error bars denote the Standard Error of the Mean (SEM). Statistical analysis was performed using an unpaired Student's t-test.

Table S1. Primary and secondary antibodies used for immunofluorescence staining (IF) and western blotting (WB).

Antibody	Supplier	Host	Dilution	Application
Lamin A/C (Jol2) monoclonal antibody	In-house	Mouse	1:20<br>1:40	IF<br>WB
Nesprin 2 (K20-478) monoclonal antibody	In-house[2]	Mouse	1:50	IF
Nesprin 2 (pAbK1) polyclonal antibody	In-house[3]	Rabbit	1:20; 1:100 (tissue)<br>1:1,500	IF<br>WB
Nesprin 2 polyclonal antibody	Abcam (ab204308)	Rabbit	1:10<br>1:250	IF<br>WB
Lamin B1 polyclonal antibody	Abcam (ab16048)	Rabbit	1:50	IF
Emerin polyclonal antibody	Abcam (ab40688)	Rabbit	1:100<br>1:1,000	IF<br>WB
GAPDH monoclonal antibody	Proteintech (60004-1-Ig)	Mouse	1:10,000	WB
Anti-Rabbit IgG (H+L) Secondary Antibody, Alexa Fluor™ 555	ThermoFisher Scientific (A-21429)	Goat	1:400 (tissue)	IF
Anti-Rabbit IgG (H+L) Secondary Antibody, Alexa Fluor™ 568	ThermoFisher Scientific (A-1101)	Goat	1:1,000	IF
Anti-Mouse IgG (H+L) Secondary Antibody, Alexa Fluor™ 568	ThermoFisher Scientific (A-1104)	Goat	1:1,000	IF
Anti-Mouse IgG (whole molecule)-Peroxidase antibody	Sigma-Aldrich (A4416)	Goat	1:10,000	WB
Anti-Rabbit IgG (whole molecule)-Peroxidase antibody	Sigma-Aldrich (A0545)	Goat	1:5,000	WB

Table S2. Fluorescent stains and conjugates used for immunostaining.

Fluorescent stain	Supplier	Concentration	Application
**Phalloidin-FITC**	Tocris Bioscience	20 ng/ml	IF
**DAPI**	Sigma-Aldrich (D9542)	2 µg/ml	IF
